# Variability in HIV-1 transmitted/founder virus susceptibility to combined APOBEC3F and APOBEC3G host restriction

**DOI:** 10.1128/jvi.01606-24

**Published:** 2024-12-23

**Authors:** Amit Gaba, Maria Yousefi, Shreoshri Bhattacharjee, Linda Chelico

**Affiliations:** 1 Department of Biochemistry, Microbiology, and Immunology, College of Medicine, University of Saskatchewan 12371 , Saskatoon, Saskatchewan, Canada; Icahn School of Medicine at Mount Sinai, New York, New York, USA

**Keywords:** HIV, APOBEC3, transmitted/founder, restriction factor

## Abstract

Several APOBEC3 enzymes restrict HIV-1 by deaminating cytosine to form uracil in single-stranded proviral (−)DNA. However, HIV-1 Vif counteracts their activity by inducing their proteasomal degradation. This counteraction by Vif is incomplete, as evidenced by footprints of APOBEC3-mediated mutations within integrated proviral genomes of people living with HIV-1. The relative contributions of multiple APOBEC3s in HIV-1 restriction are not fully understood. Here, we investigated the activity of co-expressed APOBEC3F and APOBEC3G against HIV-1 Subtype B and Subtype C transmitted/founder viruses. We determined that APOBEC3F interacts with APOBEC3G through its N-terminal domain. We provide evidence that this results in protection of APOBEC3F from Vif-mediated degradation because the APOBEC3F N-terminal domain contains residues required for recognition by Vif. We also found that HIV-1 Subtype C Vifs, but not Subtype B Vifs, were less active against APOBEC3G when APOBEC3F and APOBEC3G were co-expressed. Consequently, when APOBEC3F and APOBEC3G were expressed together in a single cycle of HIV-1 replication, only HIV-1 Subtype C viruses showed a decrease in relative infectivity compared to when APOBEC3G was expressed alone. Inspection of Vif amino acid sequences revealed that differences in amino acids adjacent to conserved sequences influenced the Vif-mediated APOBEC3 degradation ability. Altogether, the data provide a possible mechanism for how combined expression of APOBEC3F and APOBEC3G could contribute to mutagenesis of HIV-1 proviral genomes despite the presence of Vif and provide evidence for variability in the Vif-mediated APOBEC3 degradation ability of transmitted/founder viruses.

**IMPORTANCE:**

APOBEC3 enzymes suppress HIV-1 infection by inducing cytosine deamination in proviral DNA but are hindered by HIV-1 Vif, which leads to APOBEC3 proteasomal degradation. Moving away from traditional studies that used lab-adapted HIV-1 Subtype B viruses and singular APOBEC3 enzymes, we examined how transmitted/founder isolates of HIV-1 replicated in the presence of APOBEC3F and APOBEC3G. We determined that APOBEC3F interacts with APOBEC3G through its N-terminal domain and that APOBEC3F, like APOBEC3G, has Vif-mediated degradation determinants in the N-terminal domain. This enabled APOBEC3F to be partially resistant to Vif-mediated degradation. We also demonstrated that Subtype C is more susceptible than Subtype B HIV-1 to combined APOBEC3F/APOBEC3G restriction and identified Vif variations influencing APOBEC3 degradation ability. Importantly, Vif amino acid variation outside of previously identified conserved regions mediated APOBEC3 degradation and HIV-1 Vif subtype-specific differences. Altogether, we identified factors that affect susceptibility to APOBEC3F/APOBEC3G restriction.

## INTRODUCTION

The APOBEC3 (A3) enzymes belong to a family of cytosine deaminases that in humans has seven members (A3A–A3H, excluding E) (1). These enzymes act as host restriction factors against retroelements, retroviruses, and DNA viruses (2–4). APOBEC3 enzymes deaminate cytosine to uracil in single-stranded DNA replication intermediates, inducing mutations in or degradation of DNA, as well as inhibiting retroelement and retroviral reverse transcriptase through a deamination-independent mechanism (2–6).

Out of the seven human A3 family members, five (A3C S188I, A3D, A3F, A3G, and A3H [Haplotypes II, V, and VII]) have been reported to inhibit HIV-1 in the absence of its Vif protein (7–12). Vif induces degradation of A3 enzymes through the proteasome (13). In the absence of HIV Vif, APOBEC3 enzymes can become encapsidated into budding virions by binding RNAs that are also bound by HIV Gag, such as HIV genomic RNA (14, 15). When these virions infect new cells, A3 enzymes will deaminate cytosines to uracils on HIV single-stranded (−)DNA, resulting in G-to-A mutations in the (+)DNA and the creation of a non-functional provirus (4). Vif prevents this restriction activity by acting as the substrate adaptor in a Cullin Ring ligase-5 (CRL5) E3 ligase complex, which binds A3 enzymes through Vif and results in their polyubiquitination and degradation through the 26S proteasome (13). Specifically, HIV-1 Vif becomes complexed with the scaffold protein Cullin 5, Elongin C, and the co-transcription factor CBF-β (13). Cullin 5 binds Ring finger protein Rbx2, and Elongin C is an obligate dimer with Elongin B (13). This results in a six-member complex to which an A3 and ARIH2 or an E2 associates to carry out initial monoubiquitination and polyubiqitination, respectively (16). Vif requires CBF-β not directly for A3-mediated ubiquitination but for thermodynamic stability in cells, which also facilitates assembly with the CRL5 E3 ligase complex (17–19). However, counteraction of APOBEC3 by Vif is not complete, and footprints of A3-induced mutations can be found in at least 30% of integrated proviral genomes within 6 weeks of infection (20–25). At this early stage of infection, the viruses are categorized as transmitted/founder (T/F) viruses (26). Since the Vifs of T/F viruses have not been previously characterized for their ability to suppress A3-mediated restriction, the mechanism behind the accumulation of this high number of G→A mutations is not understood.

Although there are five human A3 enzymes that can restrict HIV-1, due to the *APOBEC3* genes being highly polymorphic, humans usually do not express five *APOBEC3* genes that result in enzymes able to restrict HIV (27). The least polymorphic A3 enzymes that have the highest probability to be active against HIV-1 and co-expressed are A3G and A3F (28, 29). Importantly, A3G and A3F form a hetero-oligomer that enhances HIV-1 restriction in the absence of Vif and enables the A3F in the hetero-oligomer to become partially resistant to Vif-mediated degradation in the presence of Vif (30, 31). The A3G-mediated resistance of A3F to Vif does not result in a decrease of virus infectivity in a single round of HIV-1_LAI_ replication but does result in more diversely mutated viruses that contain both A3F- and A3G-induced mutations and viruses that are less fit under selective conditions, such as anti-retroviral treatment during continuous infection in cell culture (31). The mechanism by which A3G partially protects A3F from Vif-mediated degradation is not known. Originally, alanine scanning mutagenesis was used to determine that A3G and A3F interacted with Vif using distinct domains present in the N-terminal and C-terminal domains, respectively (32–35). However, another study using single-nucleotide changes based on species-specific amino acid differences in A3F determined that the equivalent amino acid at position 128 in the A3G N-terminal domain (NTD) important for Vif-mediated degradation was also important for A3F-mediated degradation (36).

Although cryo-electron microscopy (cryo-EM) structures of Vif/CBF-β/Elongin B/Elongin C have been determined for A3G and A3H Haplotype II, there is no structural information for how full-length A3F interacts with Vif, thus requiring a dependence on mutagenesis studies to be maintained (37–40). A cryo-EM of the A3F C-terminal domain (CTD) with Vif/CBF-β determined that the Vif and CBF-β interface forms a wedge in which the A3F CTD interacts with both proteins (41). The absence of a structure with full-length A3F interacting with Vif has hindered our understanding of how the A3F/A3G hetero-oligomer results in partial resistance of A3F to Vif-mediated degradation.

In this study, we examined both the mechanism by which A3F resists Vif-mediated degradation in the presence of A3G and if this occurs across a panel of HIV-1 T/F viruses. In addition, we assessed if the partial resistance of A3F to Vif could cause a decrease in infectivity in a single round of HIV-1 replication in the absence of selective conditions. We identified that A3F interacts with A3G through its NTD and confirmed that the NTD of A3F is required for Vif-mediated degradation, suggesting an A3G-induced protection mechanism from Vif-mediated degradation. We determined that this occurred not only with HIV-1_LAI_ but also with all nine different HIV-1 T/F viruses from Subtypes B and C. Notably, the T/F viruses from Subtype B were similar to HIV-1_LAI_ and confirmed previous results that increased A3F levels cannot cause increased restriction of HIV-1 in a single replication cycle (31). However, in the presence of Subtype C HIV-1 T/F viruses, the A3F/A3G hetero-oligomer afforded protection to both A3F and A3G from Vif-mediated degradation. This enabled the A3F/A3G hetero-oligomer to cause increased restriction of Subtype C HIV-1 T/F viruses in a single replication cycle. In addition, there were differences in the capability of HIV-1 T/F Vifs to induce degradation of the A3F/A3G hetero-oligomer despite previously identified interaction domains being conserved. Rather, we determined that regions adjacent to these conserved domains determined differences in HIV-1 T/F Vif-induced degradation, particularly amino acids at positions 155 and 177. Altogether, our data redefine the Vif interaction domain for A3F and for the first time characterize the HIV-1 T/F virus Vif-mediated degradation of A3G and A3F.

## RESULTS

### A3G protects A3F from HIV-1_LAI_ Vif-mediated degradation

A3G and A3F interact through a protein-protein interaction in the absence of an RNA intermediate, and this results in partial protection of A3F from Vif-mediated degradation, but the mechanism is not known (30, 31). We studied the A3F/A3G hetero-oligomer using a transfection-based system and a plasmid with two multiple cloning sites (MCS) so that each cell that expresses A3F will also express A3G. The A3F and A3G are uniquely tagged with V5 and HA, respectively, so that they can be identified by immunoblotting. While this precludes comparison between A3G and A3F, we can compare changes among each A3 when it is expressed alone or together to interpret effects of the co-expression. Transfection also enables comparison of only A3G and A3F since primary cells express additional A3 enzymes that would preclude a mechanistic analysis of select family members (28, 42).

**Fig 1:**
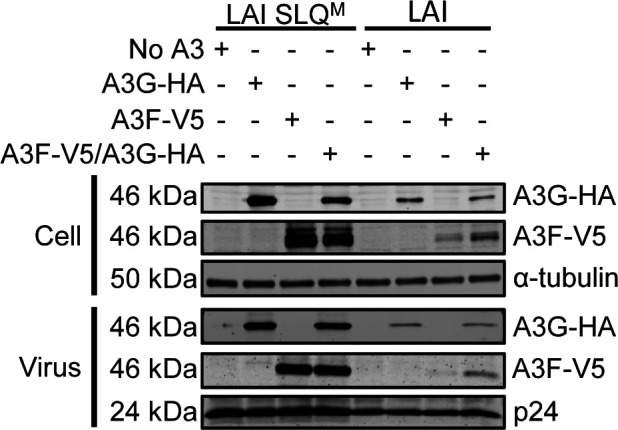
The A3F and A3G interaction results in partial protection of A3F from HIV-1_LAI_ Vif-mediated degradation. HIV-1_LAI_ (LAI) and LAI SLQ mutant (SLQ^M^) viruses were used to determine the Vif-mediated degradation of A3F and A3G. The SLQ^M^ is SLQ→AAA and prevents Vif from interacting with Elongin C. Immunoblotting with anti-HA and anti-V5 was used to detect A3G and A3F, respectively, in cell and virus lysates when each was expressed alone (A3G-HA or A3F-V5) or co-expressed (A3F-V5/A3G-HA). The no A3 condition used empty vector as a transfection control. The anti-α-tubulin and anti-p24 were used as loading controls for cell and virus lysates, respectively.

Samples from HIV-1_LAI_ producer cells transfected with A3F-V5, A3G-HA, or A3F-V5/A3G-HA were immunoblotted to detect steady-state protein levels in cell lysates (Fig. 1, cell). Previous findings using the VSV-G pseudotyped virus that showed co-expression of A3G and A3F protects A3F from Vif-mediated degradation were confirmed with a full-length molecular clone of HIV-1_LAI_ (Fig. 1, cell). That Vif is responsible for this degradation is demonstrated by the HIV-1_LAI_ SLQ^M^ that has the Vif SLQ motif needed to interact with Elongin C mutated to AAA (Fig. 1, LAI SLQ^M^). This molecular clone expresses Vif, but it cannot form a CRL5 E3 ubiquitin ligase complex. The HIV-1_LAI_ SLQ^M^ condition represents the total possible amount of steady-state A3 levels in cells (Fig. 1, cell). The data demonstrated that Vif-induced degradation by HIV-1_LAI_ of A3F occurs when it is expressed alone (by comparison to HIV-1_LAI_ SLQ^M^), but when it is co-expressed with A3G, the A3F is more resistant to Vif-mediated degradation (Fig. 1, cell).

The differences in the levels of A3F when co-expressed with A3G compared to when expressed alone were also apparent in HIV-1_LAI_ virions. The additional A3F in cells in the A3F/A3G condition resulted in increased A3F encapsidation into HIV-1_LAI_ virions (Fig. 1, virus). However, the amount of A3G encapsidated into HIV-1_LAI_ virions was similar in the absence or presence of A3F (Fig. 1, virus). Altogether, these data demonstrate that the increased steady-state level of A3F in cell lysates where A3G is also present results in increased encapsidation of A3F and is due to protection from Vif-mediated degradation.

### A3F interacts with A3G through the N-terminal domain

**Fig 2:**
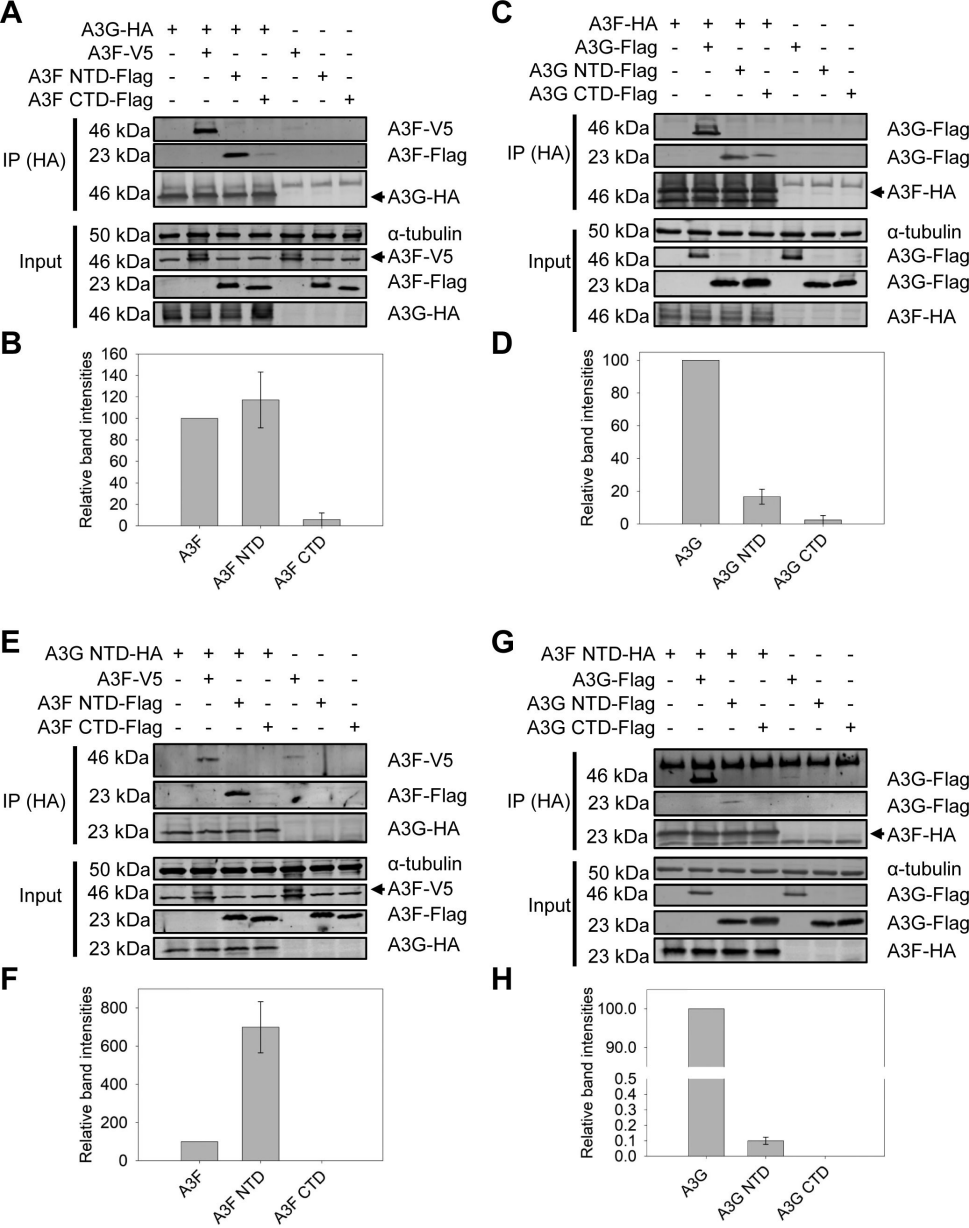
A3F interacts with A3G through the N-terminal domain. (**A and B**) Cell lysates from cells co-transfected with expression plasmids for A3G-HA and A3F-V5, A3F NTD-Flag, or A3F CTD-Flag were immunoprecipitated with anti‐HA antibody, resolved by SDS‐PAGE and transferred to nitrocellulose membrane. Immunoblotting with anti-HA and anti-V5 or anti-Flag demonstrated that predominantly, the A3F NTD interacted with A3G. (**C and D**) Cell lysates from cells co-transfected with expression plasmids for A3F-HA and A3G-Flag, A3G NTD-Flag, or A3G CTD-Flag were immunoprecipitated with anti‐HA antibody, resolved with SDS‐PAGE, and transferred to nitrocellulose membrane. Immunoblotting with anti-HA and anti-Flag demonstrated that predominantly, the A3G NTD, but also the A3G CTD, interacted with A3F. (**E and F**) Cell lysates co-transfected with expression plasmids for A3G NTD-HA and A3F-V5, A3F NTD-Flag, or A3F CTD-Flag were immunoprecipitated with anti‐HA antibody, resolved with SDS‐PAGE, and transferred to nitrocellulose membrane. Immunoblotting with anti-HA and anti-V5 or anti-Flag demonstrated that predominantly the A3G NTD interacted with full-length A3F and A3F NTD but not A3F CTD. (**G and H**) Cell lysates co-transfected with expression plasmids for A3F NTD-HA and A3G-Flag, A3G NTD-Flag, or A3G CTD-Flag were immunoprecipitated with anti‐HA antibody, resolved with SDS‐PAGE, and transferred to nitrocellulose membrane. Immunoblotting with anti-HA and anti-Flag demonstrated that predominantly, the full-length A3G, but also the A3G NTD interacted with A3F NTD. (**A–H**) Two independent experiments were conducted. A representative blot is shown. The co-IP immunoblots were analyzed to determine the band intensities of proteins in the co-IP by normalizing each to the immunoprecipitated protein in same sample. The values were converted to relative band intensities by setting the band intensity of the full-length protein in the co-IP to 100%. Data from two independent experiments were graphed with error bars representing the standard deviation.

Since the A3F/A3G hetero-oligomer results in protection of A3F from HIV-1_LAI_ Vif-mediated degradation but not A3G, we hypothesized that the A3F-A3G interface occludes the residues of A3F that interact with Vif. To determine where A3F and A3G interacted and if this overlapped with the A3F and Vif interface, we used domain constructs of A3F and A3G. A3F and A3G are double zinc deaminase domain (ZDD) enzymes, and the two ZDD domains can be stably expressed independently since they are connected by a linker region (43). We used co-immunoprecipitation (co-IP) to determine if full-length A3G-HA or A3F-HA interacted with constructs of the NTD or CTD of A3F or A3G, respectively (Fig. 2A). We found that the A3G similarly immunoprecipitated the full-length A3F and the A3F NTD but immunoprecipitated the A3F CTD 17-fold less (Fig. 2A and B). These data suggest that A3G preferentially interacts with the A3F NTD. To test the reverse scenario, A3F was used to determine the ability to immunoprecipitate A3G, A3G NTD, or A3G CTD constructs (Fig. 2C). A3F-HA immunoprecipitated full-length A3G and both the A3G NTD and A3G CTD (Fig. 2C and D). However, the amount of full-length A3G in the co-IP was ~6-fold more than the A3G NTD and ~42-fold more than the A3G CTD (Fig. 2D). This suggests that regions comprising both the A3G NTD and A3G CTD interact with A3F (Fig. 2C and D).

To conclusively determine the preferential domain for A3F and A3G interactions, we generated differentially tagged constructs of A3G NTD and A3F NTD for use in co-IP with A3F and A3G NTD and CTD domain constructs. Consistent with the full-length A3G co-IP results, the A3G NTD was able to co-IP full-length A3F and A3F NTD but not the A3F CTD (compare Fig. 2A and E). However, the amount of A3F NTD that the A3G NTD immunoprecipitated was ~7-fold more than the full-length A3F, confirming that the A3F NTD preferentially interacts with the A3G NTD (Fig. 2F). We also determined the ability of the A3F NTD to co-IP full-length A3G, A3G NTD, and A3G CTD (Fig. 2G). The results showed that although the A3F NTD could immunoprecipitate the full-length A3G and A3G NTD, the amount of A3G NTD in the co-IP was 1,000-fold less than full-length A3F (Fig. 2H). The A3F NTD was not able to co-IP the A3G CTD (Fig. 2H). These data show that the A3G NTD is necessary but not sufficient to interact with A3F NTD. Consistent with the full-length A3F co-IP data, these data support that regions comprising both the A3G NTD and A3G CTD are required for interaction with A3F (compare Fig. 2C and G).

To determine if the A3F-A3G interface occludes the residues of A3F that interact with Vif, we considered the known Vif interaction domains. The A3G NTD has been shown to interact with Vif; however, current structural and mutation data with A3F support that the CTD interacts with Vif (33, 41). The co-IP data support that the A3F-A3G interface is primarily an A3F NTD-A3G interface. As a result, the co-IP data in combination with the originally identified A3F-Vif interface do not fully explain how A3G could protect A3F from Vif-mediated degradation since the A3F CTD is not the major interface interacting with A3G.

### A3F NTD is a determinant in Vif-mediated degradation

Although previous structural and biochemical studies have reported that the A3F CTD interacts with Vif and CBF-β, these data resulted from alanine scanning mutagenesis or cryo-EM of only the A3F CTD (33, 41). However, even the original study showed that an A3F NTD-A3G CTD chimera interacted with Vif through the A3F NTD, and structural models show that the A3F NTD may interact with the Vif α1 helix (33, 44). In addition, a single point mutation at A3F amino acid 128, the equivalent amino acid that is a major determinant in the A3G and Vif interaction, can disrupt Vif-mediated degradation of A3F (36). Collectively, these data suggest a previously unidentified key determinant in the A3F NTD for Vif-mediated degradation. As a result, the A3F NTD-A3G interaction may result in occlusion of the A3F residues needed for Vif to efficiently induce A3F degradation, resulting in partial resistance.

**Fig 3:**
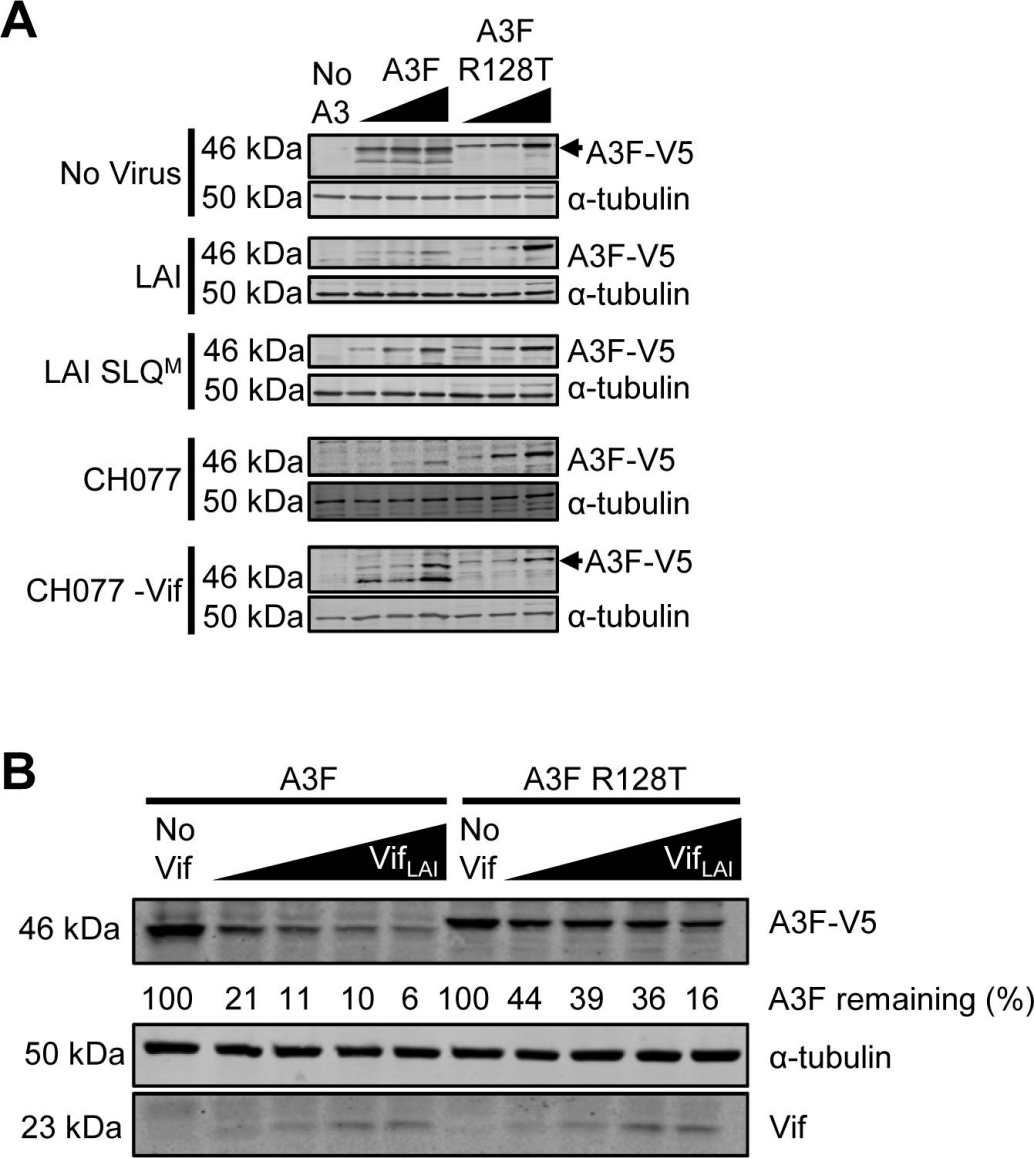
The A3F NTD is a determinant for Vif-mediated degradation. (**A**) Cells were transfected with molecular clones of HIV-1_LAI_, HIV-1_LAI_ SLQ^M^, HIV-1 T/F CH077, or HIV-1 CH077-Vif and increasing amounts of A3F-V5 (50, 100, or 200 ng) or A3F-V5 R128T (100, 200, or 300 ng) expression plasmid. Cell lysates were collected at 48 h, resolved by SDS-PAGE, transferred to nitrocellulose membrane, and probed with anti-V5 and anti-α-tubulin antibodies. The α-tubulin served as the loading control. (**B**) Cells were transfected with a constant amount of A3F-V5 (100 ng) or A3F-V5 R128T (200 ng) expression plasmid. These transfected plasmid amounts resulted in similar steady-state expression levels in cells as determined by immunoblot quantification (see Materials and Methods). Increasing amounts of HIV-1_LAI_ Vif expression construct (0, 50, 100, 200, or 300 ng) enabled assessment of the sensitivity to Vif-mediated degradation. Cell lysates were collected at 48 h, resolved by SDS-PAGE, transferred to nitrocellulose membrane, and probed with anti-V5 and anti-Vif antibodies with α-tubulin serving as loading control. The blot was analyzed to determine the band intensity of the A3 with each normalized to the α-tubulin loading control in the same sample. The values were converted to A3F remaining by normalizing to 0 ng of transfected Vif_LAI_ condition, which was set to a value of 100%. (**A and B**) Two independent experiments were conducted and showed similar results. Immunoblots are shown in this figure and Fig. S1.

To confirm and extend past studies, we used the A3F R128T mutant that showed increased restriction of HIV-1 T/F CH077 compared to A3F (36). We compared the HIV-1_LAI_ and HIV-1 T/F CH077 Vif-mediated degradation of A3F and A3F R128T (Fig. 3A). We measured the steady-state levels of A3F and A3F R128T in producer cells of HIV-1_LAI_ SLQ^M^ and HIV-1 T/F CH077 -Vif. The HIV-1_LAI_ SLQ^M^ and TF077 -Vif producer cells demonstrated similar steady-state expression levels of A3F and A3F R128T across three transfection conditions (Fig. 3A). Nonetheless, for both HIV-1_LAI_ and HIV-1 T/F CH077, the data showed that A3F R128T is more resistant to Vif-mediated degradation than A3F (Fig. 3A). That Vif is responsible for the lowered steady-state levels of the A3F is demonstrated by the HIV-1_LAI_ SLQ^M^ and TF077 -Vif producer cells that do not affect the A3F or A3F R128T steady-state levels in cells (Fig. 3A).

We also conducted an experiment with a Vif expression plasmid and transfected increasing amounts of Vif into cells with a constant amount of A3F or A3F R128T (Fig. 3B). In the absence of Vif, we quantified similar steady-state levels of A3F and A3F R128T. With increasing amounts of Vif _LAI_ transfected, there were increasing amounts of degradation of A3F and A3F R128T; however, at maximum Vif _LAI_ amounts, the amount of A3F R128T remaining was ~3-fold more than A3F (Fig. 3B). At the lowest Vif transfection condition, the A3F R128T remaining was ~2-fold more than A3F (Fig. 3B). Thus, the A3F R128T mutant is more resistant to Vif-mediated degradation than A3F. Taken together, these data demonstrate that the A3F NTD is important for Vif-mediated degradation of A3F, and amino acid 128 is a key residue. As a result, the A3G likely occludes this region of A3F, making the Vif-mediated degradation less efficient.

### A3G partially protects A3F from HIV-1 T/F Vif-mediated degradation

**Fig 4:**
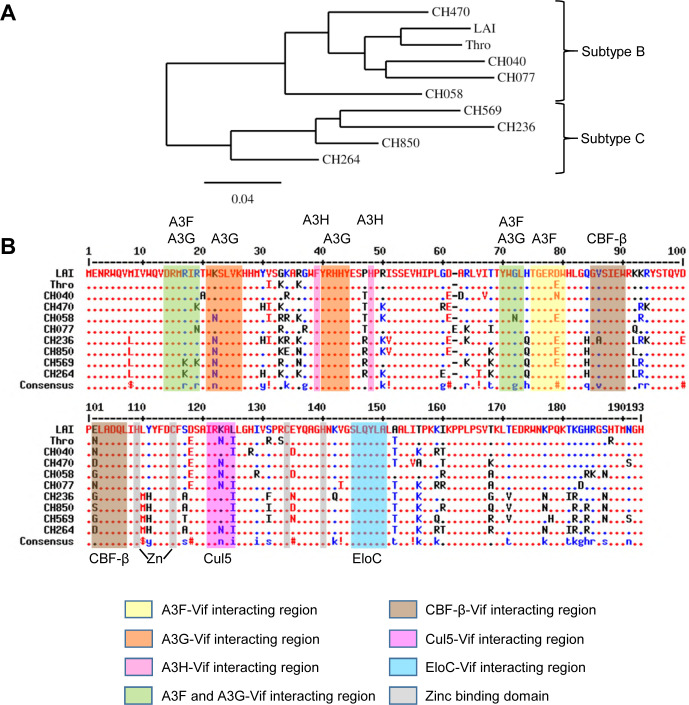
Phylogeny and amino acid alignment of HIV-1 T/F Vifs. (**A**) Phylogenetic analysis of Vif amino acid sequences of TF viruses used in this study was done using the web server Phylogeny.fr (45). (**B**) Alignment of Vif amino acid sequences from HIV-1_LAI_ and HIV-1 T/F viruses used in this study. HIV-1_LAI_ was used as the consensus Vif sequence (top). Dots indicate exact amino acid identity; amino acids indicate differences; and dashes indicate gaps introduced to optimize the alignment. Red indicates high consensus; blue indicates low consensus; and black is neutral. The symbols in the consensus sequence indicate the following amino acids: !, I and V; $, L and M; and #, B, D, E, N, Q, and Z, where B is D or N, and Z is E or Q. Known Vif interaction motifs are indicated and were obtained from references (46–61). Alignment was done with MultAlin (62). Cul5, Cullin 5; EloC, Elongin C; Zn, zinc-binding domain.

To determine if the co-expression of A3F and A3G resulted in partial protection of A3F from Vif expressed from multiple HIV-1 molecular clones, we tested nine different HIV-1 T/F viruses from Subtype B (CH058, CH470, CH040, CH077, and Thro) and Subtype C (CH236, CH264, CH850, and CH569) alongside HIV-1_LAI_. Analysis of amino acid sequences from HIV-1 T/F Vifs revealed on average 12% variation relative to the Vif of HIV-1_LAI_ (Fig. 4A). The maximum variation was 19% for CH236, and the minimum variation was 7% for T/F Thro (Fig. 4A). Further inspection of the sequences revealed that the amino acid sequence of the domains reported to be relevant for A3-Vif interactions was conserved, and only intervening regions contained the variable amino acids (Fig. 4B). Thus, we hypothesized that all the T/F Vifs of Subtype B or Subtype C would have similar degradation profiles for A3G, A3F, and A3F/A3G when compared to LAI Vif.

**Fig 5:**
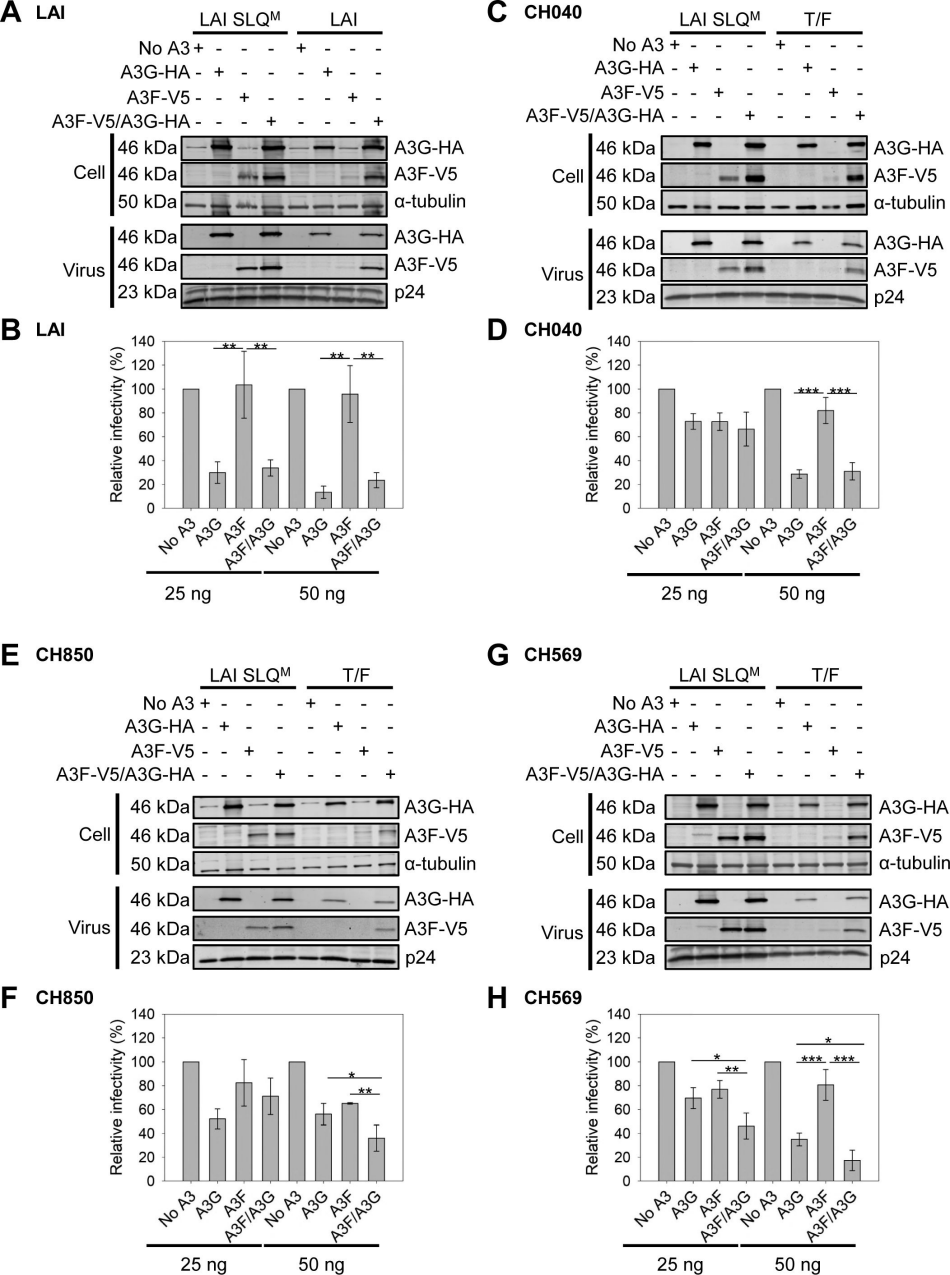
HIV-1 T/F viruses have variable infectivity in the presence of A3F, A3G, and A3F/A3G. 293T cells were co-transfected with no A3 (empty vector), A3G-HA (A3G), A3F-V5 (A3F), or A3F-V5/A3G-HA (A3F/A3G) expression plasmid and a molecular clone of (**A and B**) HIV-1_LAI_ (**C and D**) HIV-1 T/F CH040 (**E and F**) HIV-1 T/F CH850, or (**G and H**) HIV-1 T/F CH569. For immunoblots, cell lysates and virus lysates were collected at 48 h, resolved by SDS-PAGE, and transferred to nitrocellulose membrane for probing with anti-V5 and anti-HA. The HIV-1_LAI_ SLQ^M^ was used as a no-Vif control. The anti-α-tubulin and anti-p24 served as loading control for cell and virus lysate, respectively. For immunoblots, 50 ng of A3 expression plasmid was used. Immunoblots were produced from two independent experiments and showed similar results. Immunoblots are shown in this figure and Fig. S2. The relative infectivity was determined using relative β-galactosidase activity from infected TZM-bl cells normalized to the no-A3 condition. Viruses used to infect TZM-bl cells were produced from cells transfected with 25 or 50 ng of A3 expression plasmid. Error bars represent the standard deviation from three independent experiments. Designations for significant differences between values were determined using analysis of variance and are shown as *P ≤ 0.05, **P ≤ 0.01, or ***P ≤ 0.001.

We selected three HIV-1 T/F molecular clones to study in more depth using single-cycle infectivity assays. The HIV-1 T/F CH040 was used alongside HIV-1_LAI_ to represent Subtype B viruses. The HIV-1 T/F CH850 and CH569 were used to represent Subtype C viruses. We transfected either 25 or 50 ng of plasmids expressing the A3G alone, A3F alone, or A3F/A3G together with the full-length molecular clones and determined the relative infectivity in comparison to a no-A3 transfection and detected the A3s in producer cells and virions by immunoblotting (Fig. 5). The HIV-1_LAI_ SLQ^M^ was used for comparison in each experiment to determine the amount of A3 enzymes in producer cells and viruses in the absence of Vif-mediated degradation and as an experimental control for maximum A3 restriction (Fig. 5).

Although we observed protection of A3F by A3G in the presence of HIV-1_LAI_ by immunoblotting (Fig. 1 and 5A), the co-expressed A3F and A3G were not able to restrict HIV-1_LAI_ more than A3G alone, similar to what was reported previously (Fig. 5B) (31). The A3G remaining in the cells when alone or co-expressed with A3F was encapsidated (Fig. 5A, virus). This was in contrast to A3F that was not detectable in virions by immunoblotting when expressed alone but was detectable in virions when co-expressed with A3G (Fig. 1 and 5A, virus). However, when determining the infectivity, we observed that HIV-1_LAI_ was restricted similarly when A3G was expressed alone and when expressed with A3F (Fig. 5B). Mohammadzadeh et al. found similar results for infectivity but also determined that there was an effect of the A3F/A3G hetero-oligomer upon examining the mutations in proviral sequences (31). The HIV-1_LAI_ was sensitive to restriction when A3G was expressed, despite the presence of Vif and at both 25- and 50-ng transfection conditions. HIV-1_LAI_ was not sensitive to A3F-mediated restriction, consistent with previously reported lower activity of A3F and the increased sensitivity to Vif-mediated degradation (Fig. 5A and B) (29, 31). However, the HIV-1 T/F viruses showed different infectivity results from HIV-1_LAI_, although the immunoblots were similar.

Specifically, immunoblotting of producer cell and virus lysates from Subtype B HIV-1 T/F CH040 single-cycle infectivity experiments showed modest A3G degradation when alone or co-expressed with A3F and efficient A3F degradation when alone, but not when co-expressed with A3G (Fig. 5C, cell). The A3s that were observed in the cell lysates (A3G alone, A3G co-expressed with A3F, and A3F co-expressed with A3G) were encapsidated (Fig. 5C). Despite there being a significant protection of A3F from Vif-mediated degradation and encapsidation of A3F in the presence of A3G, the infectivity data showed minimal effect of A3G alone, A3F alone, or A3F/A3G on T/F CH040 when only 25 ng of the A3s was transfected (Fig. 5C and D). When the transfection level of the A3s was doubled, we observed A3G, but not A3F restriction when both were expressed alone. The effect of A3F/A3G co-expression was similar to A3G alone (Fig. 5D).

The HIV-1 Subtype C T/F viruses CH850 and CH569 showed different infectivity results from the Subtype B viruses. Interestingly, the immunoblots for both HIV-1 T/F CH850 and CH569 were similar to those of the Subtype B viruses and showed modest A3G degradation when alone or co-expressed with A3F, efficient degradation of A3F when expressed alone, and protection of A3F from Vif-mediated degradation when co-expressed with A3G (Fig. 5E and G). For HIV-1 T/F CH850, the infectivity was not affected by expression of A3G alone, A3F alone, or A3F/A3G at 25-ng plasmid transfection. However, at the 50-ng plasmid transfection, we observed no difference in the sensitivity to A3G or A3F alone, but there was ~1.5-fold more sensitivity to A3F/A3G compared to A3G alone, resulting in only ~35% remaining infectivity (Fig. 5F, *P* < 0.05). There was even more of an effect of co-expression of A3F/A3G for HIV-1 T/F CH569 where we observed ~1.5- to 2.0-fold more reduction in infectivity for A3F/A3G compared to A3G alone at both the 25- and 50-ng plasmid transfections with only ~20% infectivity remaining at the higher A3F/A3G transfection amount (Fig. 5H, *P* < 0.05). These effects are noteworthy, considering that Vif expression is occurring from these molecular clones.

Overall, we observed similar results for other Subtype B and C HIV-1 molecular clones (Fig. S3 to S5). The Subtype B molecular clones were more sensitive to restriction by A3G when expressed alone and showed no difference from A3G alone for the restriction in the A3G/A3F condition (Fig. S3 and S5). The Subtype B molecular clones also showed a large variability in sensitivity to A3G and A3F/A3G, with infectivity ranging from 13% to 60% in the presence of A3G and from 18% to 66% in the presence of A3F/A3G (Fig. S3). The Subtype C molecular clones were less sensitive to restriction by A3G when expressed alone but showed an approximately 1.4- to 2.0-fold reduction in infectivity in the presence of A3F/A3G in comparison to A3G alone (Fig. S4). The T/F CH236 was most similar to T/F CH569 and had a significant reduction in infectivity in the presence of A3F/A3G compared to A3G alone (Fig. S4C through D; *P*<0.01). None of the molecular clones were sensitive to restriction by A3F when it was expressed alone (Fig. S3 and S4).

### HIV-1 T/F Subtype C-specific differences in A3G-mediated degradation

**Fig 6:**
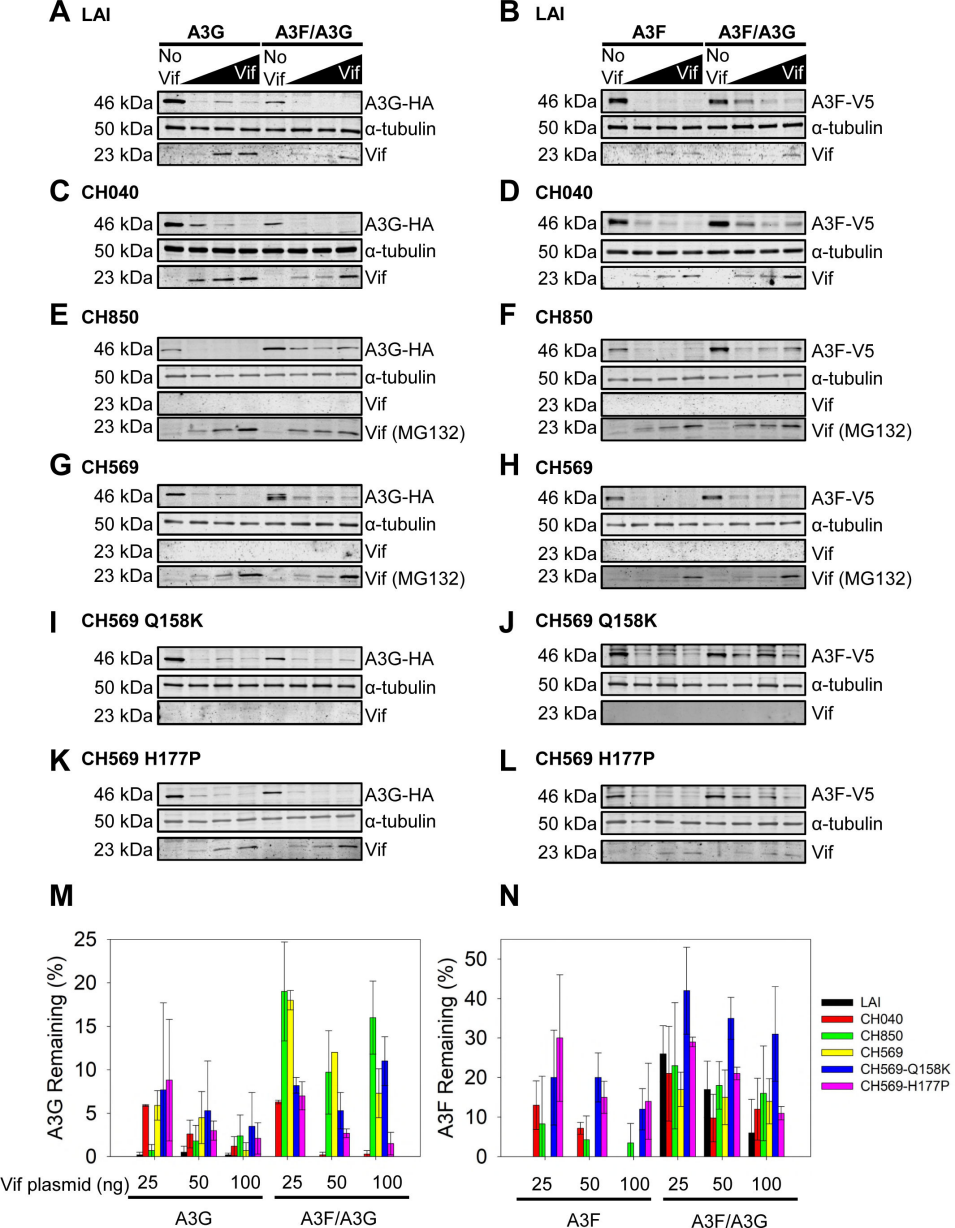
Vif-mediated degradation of A3F, A3G, and A3F/A3G differs between HIV-1 Subtype B and Subtype C viruses. 293T cells were co-transfected with plasmid expressing either A3G-HA (A3G), A3F-V5 (A3F), or A3F-V5/A3G-HA (A3F/A3G) and increasing amount of Vif-AU1 expression plasmid (0, 25, 50, and 100 ng) from HIV-1 (**A and B**) LAI, (**C and D**) T/F CH040, (**E and F**) T/F CH850, (**G and H**) T/F CH569, (**I and J**) T/F CH569 Q158K, and (**K and L**) T/F CH569 H177P. Cell lysates were collected at 44 h, resolved by SDS-PAGE, and transferred to nitrocellulose membrane for probing with anti-V5, anti-HA, and anti-Vif antibody. The anti-α-tubulin was the loading control. Two independent experiments were conducted and showed similar results. Immunoblots are shown in this figure and Fig. S6. (E–H, bottom immunoblot) Cells were treated with MG132 (a proteasome inhibitor) for 14 h before collection of cell lysate to demonstrate that Subtype C Vifs have a propensity to be degraded with the bound A3 but were present in the cells upon transfection. (**A–L**) Two independent experiments were conducted. A representative blot is shown. (**M and N**) The immunoblots were analyzed to determine the band intensities of A3G or A3F remaining by normalizing to the α-tubulin in each respective lane. The values were converted to relative band intensities by setting the band intensity of the A3 from the No Vif condition to 100%. Data from two independent experiments were graphed with error bars representing the standard deviation.

Despite clear trends emerging from the infectivity data and corresponding immunoblots, we wanted to conduct a more quantitative assay to characterize the potential differences in the Vif-mediated degradation of Subtype B and Subtype C T/F virus Vifs. To this end, we cloned the *vif* from HIV-1_LAI_ and the T/F viruses CH040, CH850, and CH569. We used these clones for A3 degradation assays using co-transfection of a constant amount of A3G, A3F, or A3F/A3G expression plasmid and increasing amounts of Vif expression plasmid (0, 25, 50, and 100 ng). For the Subtype B Vifs from HIV-1_LAI_ and T/F CH040, there was less A3G remaining in the A3F/A3G condition in comparison to Subtype C virus Vifs (Fig. 6A, C, E, G and M). Specifically, for T/F CH040, there was threefold less A3G in the A3F/A3G condition when compared to CH850 and CH569 (Fig. 6C, E, G and M). This suggests that HIV-1 Subtype B Vifs are more efficient than Subtype C viruses in causing degradation of A3G when it is co-expressed with A3F. There was no consistent subtype differences in the ability of Vif to induce degradation of A3G when expressed alone. Interestingly, the Subtype C virus Vifs were not detectable on the blots and were presumably degraded with the A3, in contrast to the Subtype B Vifs that could be detected by immunoblotting (Fig. 6A through H). To test this directly, we conducted a parallel experiment where cells transfected with the Subtype C viruses were treated with the proteasome inhibitor MG132. We were able to detect the Subtype C virus Vifs by immunoblotting when proteasomal degradation was inhibited (Fig. 6E through H, Vif [MG132]), confirming that Subtype C virus Vifs were degraded with the A3. Additionally, we observed that for all T/F viruses tested, there was more A3F remaining in the A3F/A3G condition compared to when A3F was expressed alone, consistent with blots from infectivity assays (compare Fig. 5, 6B, D, F, H, and N). Altogether, these data suggest that the HIV-1 T/F Subtype C viruses are more sensitive to the A3F/A3G hetero-oligomer than HIV-1 T/F Subtype B because the Vifs are less able to induce degradation of A3G (Fig. 6A, C, E and G).

### Amino acid determinants for Vif-mediated degradation occur outside of conserved domains

The Vif domains for interaction with CBF-β, Elongin C, and Cullin 5 are well conserved since deviation from these motifs would disrupt function (Fig. 4B). Nonetheless, we examined the amino acid alignment of the HIV-1 T/F Vifs to determine if we could identify any amino acids in Subtype C in comparison to Subtype B viruses that account for differential abilities of the Vifs to induce A3 degradation (Fig. 4B). All analyzed Subtype C viruses but none of the Subtype B viruses had a leucine at position 8 that is near the DRMR A3F-A3G interaction domain, a histidine at position 83 and leucine at position 91 near the CBF-β interaction domain, and a methionine and histidine at 110–111 near the CBF-β and Zn^2+^ interaction domains (Fig. 4B).

While all these amino acids likely contribute individually or collectively to the differences we observed with infectivity and A3-induced degradation, we focused on CH569 since it had a unique profile for infectivity (Fig. 5). The CH569 was most sensitive to the A3F/A3G co-expression, and this appeared to be due to a lesser ability to induce degradation of A3G, in addition to an inability to induce full degradation of A3F in the A3F/A3G hetero-oligomer (Fig. 5H, 6G and H). We observed that CH569 Vif had two unique amino acids from all other Subtype B and Subtype C Vifs that we tested (Fig. 4B). We mutated these amino acids to become more like the other T/F Vifs and LAI Vif, resulting in CH569 Q158K and CH569 H177P Vifs.

We used these CH569 mutant Vifs in an A3 degradation assay. When A3F was co-expressed with A3G, ~3-fold more A3F was remaining for the CH569 Q158K Vif mutant and ~2-fold more A3F was remaining for the CH569 H177P Vif mutant compared to CH569 Vif (Fig. 6H, J, L and N; 25-ng Vif plasmid transfected). This suggests that these amino acids are required for efficient degradation of A3F in the A3F/A3G hetero-oligomer. These mutations were predicted to make CH569 more Subtype B-like, and the results align well with our HIV-1 Subtype B data, where more A3F was remaining in the A3F/A3G expression condition compared to when A3F was expressed alone. For CH569 Q158K and CH569 H177P, there was also less degradation of A3F alone compared to wild-type CH569 (Fig. 6H, J, L and N). Interestingly, when A3G was expressed alone, CH569 Vif and the CH569 Vif mutants caused a similar level of A3G degradation (Fig. 6G, I, K and M). However, in the A3F/A3G condition, both the CH569 H177P and CH569 Q158K mutants were ~2- to 3-fold more efficient than wild-type CH569 at inducing degradation of A3G (Fig. 6G, I, K and M; 25- and 50-ng Vif plasmid transfected) and more similar to Vif-mediated degradation observed for Subtype B viruses, as would be predicted by the amino acid substitutions (Fig. 6A and C; 25- and 50-ng Vif plasmid transfected). Overall, these data suggest that there are still unidentified regions of Vif that are determinants in A3-mediated degradation. This is especially important for understanding the A3F and A3F-A3G interactions with Vif since there is no structural information for full-length A3F in complex with Vif.

## DISCUSSION

A3 restriction factors are able to induce mutations in HIV-1 proviral DNA despite Vif-mediated degradation. Near full-length sequencing of HIV-1 proviral DNA within the acute infection period has shown that ~95% of proviral DNA becomes inactivated within the first 6 weeks of infection, and at least 30% of those proviruses have A3-induced G→A mutations, particularly causing stop codons that result in viral inactivation (20, 63). While this initially appeared at odds with a functional Vif protein, A3F and A3G have been shown to hetero-oligomerize and co-encapsidate in order to partially resist Vif-mediated degradation, enabling their activity to tip the balance toward viral inactivation (30, 31, 64). We have previously shown that in the presence of Vif and anti-retrovirals, A3F/A3G hetero-oligomerization results in partial protection of A3F from Vif-mediated degradation, increased proviral mutations from A3F, and greatly decreased viral fitness compared to the absence of A3s (31). However, how A3F and A3G interacted and if these identified hetero-oligomer features occurred in the presence of HIV-1 T/F Vifs were not known. Here, we determined that the A3F NTD interacts with A3G, confirmed and extended data that suggested the A3F NTD is a determinant in Vif-mediated degradation, determined that multiple HIV-1 T/F viruses lose the ability to fully induce degradation of A3F when it hetero-oligomerizes with A3G, and identified new determinants in Vif-mediated degradation outside of the previously identified domains.

The co-IP data demonstrated that the A3F NTD interacted with A3G. An additional consideration in this interaction is the stoichiometry and RNA binding. Ara et al. determined that three molecules of A3F interacted with one molecule of A3G (30). However, the orientation of the A3F interacting domains for homo-oligomerization is not known, making it difficult to model the A3F-A3G interaction and how the interaction is able to protect A3F from Vif-mediated degradation. That the NTD of A3F is involved in Vif-mediated degradation and the interaction with A3G suggests that A3G occludes the Vif interaction site. However, it does not explain why other A3F subunits of the hetero-oligomer are also protected from Vif-mediated degradation or why for HIV-1 Subtype B T/F viruses A3G is not also protected. One explanation is that both A3G hetero- and A3F homo-oligomerization block the Vif interaction site on A3F but not A3G. A3G may have a stronger binding affinity for Vif than A3F, thus causing Vif to selectively interact with A3G and simultaneously disrupt the hetero-oligomer, rescuing A3F from degradation. Notably, multiple structural studies have demonstrated that Vif interacts with a monomer of A3G and an RNA molecule (37–39). How the RNA molecule associates with the A3F/A3G hetero-oligomer and if A3F also interacts with Vif and RNA together remain to be determined. Alternatively, since the A3F protection from Vif-mediated degradation is only partial, this could mean that only the A3F interacting with A3G is protected from Vif-mediated degradation, and other A3F molecules in the hetero-oligomer are not. For HIV-1 Subtype C T/F viruses, the A3G within the A3F/A3G hetero-oligomer is also protected from Vif-mediated degradation. Thus, further comparative studies between HIV-1 Subtype B and Subtype C viruses will aid in clarifying the mechanism by which HIV-1 Subtype B T/F viruses interacting with the A3F/A3G hetero-oligomer cannot fully induce degradation of A3F but can induce degradation of A3G.

The A3F/A3G hetero-oligomer may affect Vif-mediated ubiquitination. Studies that determined where the CRL5 E3 ligase polyubiquitinates lysines on A3G and A3F found different results. Iwatani et al. determined that A3G was polyubiquitinated on four key residues, and these could be mutated to block Vif-mediated ubiquitination (65). However, Albin et al. determined that although A3G was partially resistant to Vif-mediated degradation when the four key residues identified by Iwatani et al. were mutated, it did not result in complete resistance to Vif (65, 66). These results and that A3F was found to be polyubiquitinated throughout the protein and modification of specific lysines could not block Vif-mediated degradation of A3F resulted in the conclusion that ubiquitination of A3s by the CRL5 E3 ligase is relatively stochastic (66). As a result, we hypothesize an all-or-nothing phenomenon where those A3F molecules interacting with A3G are not ubiquitinated due to not directly interacting with Vif rather than a hypothesis of less efficient ubiquitination of A3F resulting in partial protection of A3F from Vif-mediated degradation.

Combined restriction of HIV-1 from A3F and A3G in the presence of Vif has resulted in variable conclusions. An early study found that in the absence of Vif, co-expressed A3G and A3F caused an increase in HIV-1 proviral DNA mutations compared to when each was expressed alone (67). However, the effect of co-expression of A3F and A3G on infectivity was only additive (67). Although in the absence of Vif Ara et al. found that there was a synergistic effect of A3F and A3G co-expression on HIV-1 proviral mutation rate and restriction, in the presence of Vif, Mohammadzadeh et al. observed that there was no effect on either of these variables (30, 31). Rather, the effects observed by Mohammadzadeh et al. were that the proviral mutation type became more diversified and that the viruses were less fit under selective conditions, such as anti-retroviral treatment (31). These studies all used HIV-1 Subtype B viruses, and our data are in agreement with these studies showing that despite partial protection of A3F from Vif-mediated degradation, in a single cycle of infection, there is no effect on HIV-1 infectivity above that of A3G alone. There are two hypotheses for these results with HIV-1 Subtype B viruses in our study. This could be due to the lack of a selective pressure that would reveal defects in fitness or that more replication cycles are needed to see a pronounced effect in the presence of Vif. These hypotheses are not mutually exclusive. Mohammadzadeh et al. supports the former hypothesis (31). The latter hypothesis is supported by mouse studies. Krisko et al. used a BLT humanized mouse model and concluded that for consistent HIV-1 restriction, the combined activity of A3F and A3G was required (68). However, Sato et al., utilizing a NOG-hCD34 humanized mouse model, found that mutations in HIV-1 proviral DNA were caused by deamination activity of either A3G or A3F, suggesting that A3F and A3G act individually but still concluding that they can restrict HIV-1 in the presence of Vif (69).

Surprisingly, there was a consistent and different observation when A3F and A3G co-expression occurred during infection with HIV-1 T/F Subtype C viruses. The four HIV-1 T/F Subtype C viruses used in this study all had a 1.5- to 2.0-fold decrease in infectivity during A3F/A3G co-expression compared to A3G alone (50-ng A3 plasmid transfection). When compared to HIV-1 T/F Subtype B viruses, this did not result from a difference in the level of A3F protection from Vif-mediated degradation but from a lesser ability to induce degradation of A3G within the A3F/A3G hetero-oligomer. However, when A3G was expressed alone, the HIV-1 T/F Subtype B and Subtype C viruses showed a large diversity in the A3G-mediated restriction, with Subtype B viruses on average being more sensitive than Subtype C viruses to A3G-mediated restriction. However, during A3F-A3G co-expression, the HIV-1 T/F Subtype C viruses were more sensitive to restriction. These data suggest that the additional A3G encapsidated in the presence of A3F for HIV-1 T/F Subtype C viruses but not Subtype B viruses causes the more efficient restriction. However, since this does not occur when A3G is expressed alone during HIV-1 T/F Subtype C infection, the data support that there is a contribution of restriction from A3F. This may be due to the increased diversity in proviral DNA mutations as identified by Mohammadzadeh et al. (31). Since A3F deaminates the 5'TC motif in (−)DNA, it has a greater number of possible codons that this site overlaps with compared to A3G that deaminates 5′CC (underlined C primarily deaminated) and causes mutations primarily at glycines (70% of total mutations) (70).

The variability in the HIV-1 T/F virus sensitivity to A3G-mediated restriction was surprising since the A3-Vif interaction domains are highly conserved. However, outside of these regions, Vifs display variability, particularly subtype-specific amino acid variability (71–73). Although, HIV-1 Subtype C is the most prevalent subtype globally and accounts for more than 40% global infections, there are few studies using HIV-1 Subtype C Vif to map Vif-A3 interacting regions, in addition to a lack of A3-Vif studies using Vifs from HIV-1 T/F viruses. Our data indicate that Vif amino acids outside the well-defined Vif-A3 interfaces influence the degradation ability. However, the residues we identified as unique to the HIV-1 T/F CH569 Vif were in a loop region not well defined in cryo-EM or structural studies (37–39, 46). Nonetheless, this region is between the Elongin C interaction site and the PPLP motif of Vif that was previously shown to be important for A3-mediated degradation. As a result, additional structure-function studies in this region of Vif are needed.

The data presented here reveal additional details on the A3F-A3G interaction, demonstrate that HIV-1 T/F viruses have differential sensitivities to A3G and A3F/A3G restriction, that HIV-1 T/F Subtype C viruses are more sensitive than Subtype B viruses to the A3F/A3G hetero-oligomer mediated restriction, and identify new Vif amino acids involved in A3-mediated degradation. Altogether, the data emphasize the variability in A3 degradation ability between different Vifs and that additional studies of Vifs from T/F viruses, especially those of Subtype C origin, are warranted.

## MATERIALS AND METHODS

### Plasmids and molecular clones

The expression plasmids pVIVO2 A3G-HA, pVIVO2 A3F-V5, and pVIVO2 A3F-V5/A3G-HA have been previously described (30). The pcDNA3.1 A3F R128T-V5 has been previously described (36). The following reagents were obtained through the NIH HIV Reagent Program, Division of AIDS, NIAID, NIH: plasmid pcDNA3.1-APOBEC3G-HA expressing human APOBEC3G with C-terminal triple HA tag, ARP-9952, contributed by Dr. Warner C. Greene, and human APOBEC3F V5 His expression vector, ARP-10100, contributed by Dr. B. Matija Peterlin and Dr. Yong-Hui Zheng. The ARP-10100 APOBEC3F plasmid was subcloned in place of A3G in the ARP-9952 vector background. Flag-tagged constructs in pcDNA3.1 for A3F NTD, A3F CTD, A3G, A3G NTD, and A3G CTD were provided by Dr. Xiaojiang S. Chen (University of Southern California). HA-tagged constructs in pcDNA3.1 for A3G NTD and A3F NTD were generated by amplifying A3G NTD and A3F NTD sequences from pcDNA3.1-APOBEC3G-HA and pcDNA3.1-APOBEC3F-HA plasmids, respectively, and cloning into pcDNA3.1 using an XbaI site. The expression plasmid pcDNA3.1 Vif_LAI_ has been previously described (74). The pCG-Vif-AU1 plasmids were generated by PCR-amplifying Vif sequences from infectious molecular clones and inserted using XbaI and MluI sites.

The infectious molecular clone of HIV-1_LAI_ and the HIV-1_LAI_ SLQ→AAA (SLQ^M^) that contains a mutated Vif that cannot interact with Elongin C or induce degradation of A3s has been previously described (75). The infectious molecular clones for the HIV-1 T/F viruses were a gift from Dr. Beatrice Hahn (Perelman School of Medicine, University of Pennsylvania) and have been previously described (76–79). The CH077 -Vif construct has two tandem stop codons inserted in *vif* and was previously described (36).

### Co-immunoprecipitation

The 293T cells were seeded in a T75 cm^2^ flask (1 × 10^6^) and the next day were transfected with plasmid DNA using GeneJuice transfection reagent (EMD Millipore) as per manufacturer’s instructions. The following amounts of plasmid DNA were used to achieve similar steady-state protein levels in cells: pVIVO2 A3F-V5 (2 µg), pcDNA3.1 A3F NTD-Flag (3 µg), pcDNA3.1 A3F CTD-Flag (2 µg), pcDNA3.1 A3G-HA (1 µg), pcDNA3.1 A3F-HA (4 µg), pcDNA3.1 A3G-Flag (1 µg), pcDNA3.1 A3G NTD-Flag (2 µg), pcDNA3.1 A3G NTD-Flag (2 µg), pcDNA3.1 A3G NTD-HA (4 µg), and pcDNA3.1 A3F NTD-HA (2 µg). Empty pcDNA3.1 was used to equalize the amount of transfected DNA. At 48 h post transfection, the cells were washed with phosphate-buffered saline (PBS) and lysed using co-IP buffer (50-mM Tris-Cl, pH 7.4, 1% Nonidet-P40, 10% glycerol, and 150-mM NaCl) supplemented with EDTA-free protease inhibitor (Roche) and clarified by centrifugation. Then 400 µg of cell lysate was added to anti-HA magnetic beads (Sigma) in the presence of RNaseA (50 µg/mL, Roche) and incubated for 2 h with gentle rocking at 4°C. The beads were subsequently washed three times with co-IP buffer, and the immunoprecipitated proteins were eluted with Laemmli buffer and subjected to SDS-PAGE and immunoblotting with anti-Flag antibody (Cat #F1804; Sigma, 1:1,000), anti-HA antibody (Cat #H6908; Sigma, 1:10,000), and anti-α-tubulin antibody (Cat #PA1-20988; Invitrogen, 1:1,000). Secondary detection was performed using Licor IRDye antibodies produced in goat (IRDye 680-labeled anti-rabbit, 1:10,000, Cat #926–68071, and IRDye 800-labeled anti-mouse, 1:10,000, Cat #926–32210). Two independent co-IP experiments were conducted, and the immunoblot intensity of bands was determined using Image Studio. The band intensities of co-immunoprecipitated proteins were normalized to the immunoprecipitated protein for each respective sample. The values were converted to relative band intensities by setting the band intensity of co-immunoprecipitated full-length A3F or A3G to 100%.

### A3 degradation assay

To investigate the ability of Vif expressed from infectious molecular clones to induce degradation of A3F and A3F R128T, 293T cells (1 × 10^5^ per well) in 12-well plates were co-transfected with pVIVO2 A3F-V5 (50, 100, and 200 ng) or pcDNA3.1 A3F R128T-V5 (100, 200, and 300 ng) expression vectors and 500 ng of infectious molecular clones of HIV-1_LAI_, HIV-1_LAI_ SLQ^M^, HIV-1 T/F CH077, or HIV-1 T/F CH077 –Vif using GeneJuice (Novagen) transfection reagent according to manufacturer’s instructions. Empty vector was used to equalize the amount of transfected DNA. Since pVIVO2 and pcDNA3.1 use different promoters, the steady-state protein levels of A3F-V5 and A3F R128T-V5 were different if the same amount of DNA was transfected. As a result, we determined through quantification of immunoblot intensity that the pcDNA3.1 A3F R128T-V5 required twofold more transfected DNA to have similar steady-state expression levels to pVIVO2 A3F-V5 (see Results). At 16 h post transfection, the media was changed. After 44 h post transfection, cells were washed with PBS and lysed using 2× Laemmli buffer.

In order to compare the efficiency of HIV-1_LAI_ Vif in mediating the degradation of A3F and A3F R128T, 293T cells (1 × 10^5^ per well) in 12-well plates were co-transfected with 100-ng pcDNA3.1 A3F-V5 or 200-ng pcDNA3.1 A3F-V5 R128T and an increasing amount of pcDNA3.1 Vif_LAI_ (50, 100, 200, or 300 ng) using GeneJuice (Novagen) transfection reagent according to manufacturer’s instructions. Empty vector was used to equalize the amount of transfected DNA. Then, 16 h post transfection, the media was changed. At 44 h post transfection, cells were washed with PBS and lysed using 2× Laemmli buffer.

To compare efficiency of different Vifs in mediating degradation of A3G-HA, A3F-V5, and co-expressed A3F-V5/A3G-HA, 293T cells (1 × 10^5^ per well) in 12-well plates were co-transfected with 50 ng of pVIVO2 expressing no A3 (empty vector), A3G-HA, A3F-V5, or A3F-V5/A3G-HA and titration of pCG-Vif-AU1 (0, 25, 50, and 100 ng) using GeneJuice (Novagen) transfection reagent according to manufacturer’s instructions. To equalize the amount of plasmid DNA transfected, an empty pCG vector was used. Then, 16 h post transfection the media was changed. At 44 h post transfection, cells were washed with PBS and lysed using 2× Laemmli buffer. For pCG-CH850 Vif-AU1 and pCG-CH569 Vif-AU1, a parallel experiment was performed where cells were treated with the proteasome inhibitor MG132 (12.5 µM) for 14 h before collection of cell lysates.

Protein from each cell lysate (30 µg) was resolved by SDS-PAGE and used for immunoblotting on nitrocellulose membrane. Primary detection was performed using mouse monoclonal anti-HA antibody (Cat #H9658; Sigma, 1:10,000), anti-V5 mouse monoclonal antibody (Cat #V8012; Sigma, 1:1,000), rabbit anti-Vif antibody (Cat #809; NIH HIV Reagent Program, contributed by Dr. Bryan Cullen, 1:1,000), and anti-α tubulin antibody (rabbit polyclonal, Cat #PA1-20988; Invitrogen, 1:1,000). Although the cloned Vif was AU1 tagged, during the course of this study, the AU1 antibody was discontinued. To be consistent, we kept the plasmid as is but detected the Vif with the rabbit anti-Vif antibody, rather than the AU1 tag. Secondary detection was performed using Licor IRDye antibodies produced in goat (IRDye 680-labeled anti-rabbit 1:10,000, Cat #926–68071, and IRDye 800-labeled anti-mouse, 1:10,000, Cat #926–32210). For quantification, Image Studio was used to detect the pixel intensity of the experimental and loading control bands. Each sample was normalized to its own loading control before comparison to other lanes. Two independent immunoblot experiments were conducted for each condition.

### Single-cycle infectivity assay

Single-cycle infectivity assays were carried out using 1 × 10^5^ 293 T cells per well in a 12-well plate. The 293T cells were maintained in Dulbecco's modified Eagle medium with 10% FBS in the presence of 5% CO_2_ at 37°C. Transfections used 500 ng of an HIV-1 infectious molecular clone (LAI, LAI SLQ^M^, T/F CH058, T/F CH470, T/F CH040, T/F CH077, T/F Thro, T/F CH236, T/F CH264, T/F CH850, or T/F CH569) and 25 or 50 ng of pVIVO2 containing no A3 (empty vector), A3G-HA, A3F-V5, or both A3F-V5 and A3G-HA. The pVIVO2 (Invivogen) has two MCSs to enable co-expression of two genes on a single-cell basis. GeneJuice (Novagen) transfection reagent was used according to manufacturer’s instructions. At 16 h post transfection, the media was changed. Culture supernatants containing the virus were harvested 46 h post transfection, filtered through 0.45-µm polyvinylidene difluoride syringe filters, and used to infect TZM-bl cells. TZM-bl cells were plated at 1 × 10^4^ cells per well of a 96-well plate and infected with a serial dilution of the virus in the presence of polybrene (8 µg/mL). Forty-eight hours after infection, the cells were washed with PBS, and infectivity was measured through colorimetric detection using a β-galactosidase assay reagent and a spectrophotometer. Infectivity of each virus was compared by setting the infectivity of the “no-A3” condition as 100%. Three independent experiments were conducted.

### Immunoblotting cell and virus lysates

The cells and culture supernatants used for immunoblotting were obtained from the single-cycle infectivity assays. Cells were washed with PBS and lysed with 2× Laemmli buffer. Total protein in the cell lysate (30 µg) was resolved by SDS-PAGE for immunoblotting on a nitrocellulose membrane. A3 encapsidation was determined by concentrating the virus containing supernatant using Retro-X Concentrator (Clontech) following the manufacturer’s protocol, resuspending the pellet in 45 µL of Laemmli buffer and resolving 12 µL of the virus concentrate by SDS-PAGE for immunoblotting on a nitrocellulose membrane. HA-tagged proteins were detected in cell lysates using mouse monoclonal anti-HA antibody (Cat #H9658; Sigma, 1:10,000). HA-tagged proteins were detected in virus lysates using rabbit polyclonal anti-HA antibody (Cat #H6908; Sigma, 1:1,000). The V5-tagged proteins were detected in cell and virus lysates using anti-V5 mouse monoclonal antibody (Cat #V8012; Sigma, 1:1,000). Loading control for cell lysate was detected with anti-α-tubulin (rabbit polyclonal, Cat #PA1-20988; Invitrogen, 1:5,000) and that for virus lysate was detected with anti-p24 (mouse monoclonal, Cat #3537; NIH HIV Reagent Program, 1:1,000). Secondary detection was performed using Licor IRDye antibodies produced in goat (IRDye 680-labeled anti-rabbit, 1:10,000, Cat #926–68071, and IRDye 800-labeled anti-mouse, 1:10,000, Cat #926–32210). Two independent immunoblot experiments were conducted.

## Data Availability

All data are available within the article and supplemental material.

## SUPPLEMENTAL MATERIAL

Supplemental figures jvi.01606-24-s0001.pdf Figures S1 to S6.

ASM does not own the copyrights to Supplemental Material that may be linked to, or accessed through, an article. The authors have granted ASM a non-exclusive, world-wide license to publish the Supplemental Material files. Please contact the corresponding author directly for reuse.
