# Supplementary material for: Variability in HIV-1 transmitted/founder virus susceptibility to combined APOBEC3F and APOBEC3G host restriction
Source: J Virol. 2024 Dec 23;99(1):e01606-24. doi: 10.1128/jvi.01606-24 (PMC11784016; doi:10.1128/jvi.01606-24)
Supplement: Supplemental figures — Figures S1 to S6. [file jvi.01606-24-s0001.pdf]

Supporting Information

Variability in HIV-1 Transmitted/Founder Virus Susceptibility to Combined APOBEC3F and APOBEC3G Host Restriction

Amit Gaba[1*], Maria Yousefi[1], Shreoshri Bhattacharjee[1], Linda Chelico[1,*]

[1] Department of Biochemistry, Microbiology, and Immunology, College of Medicine,

University of Saskatchewan, Saskatoon, Saskatchewan, Canada, S7N 5E5.

* Corresponding authors

Emails: linda.chelico@usask.ca (LC) and amit.gaba@usask.ca (AG)

Figure S1

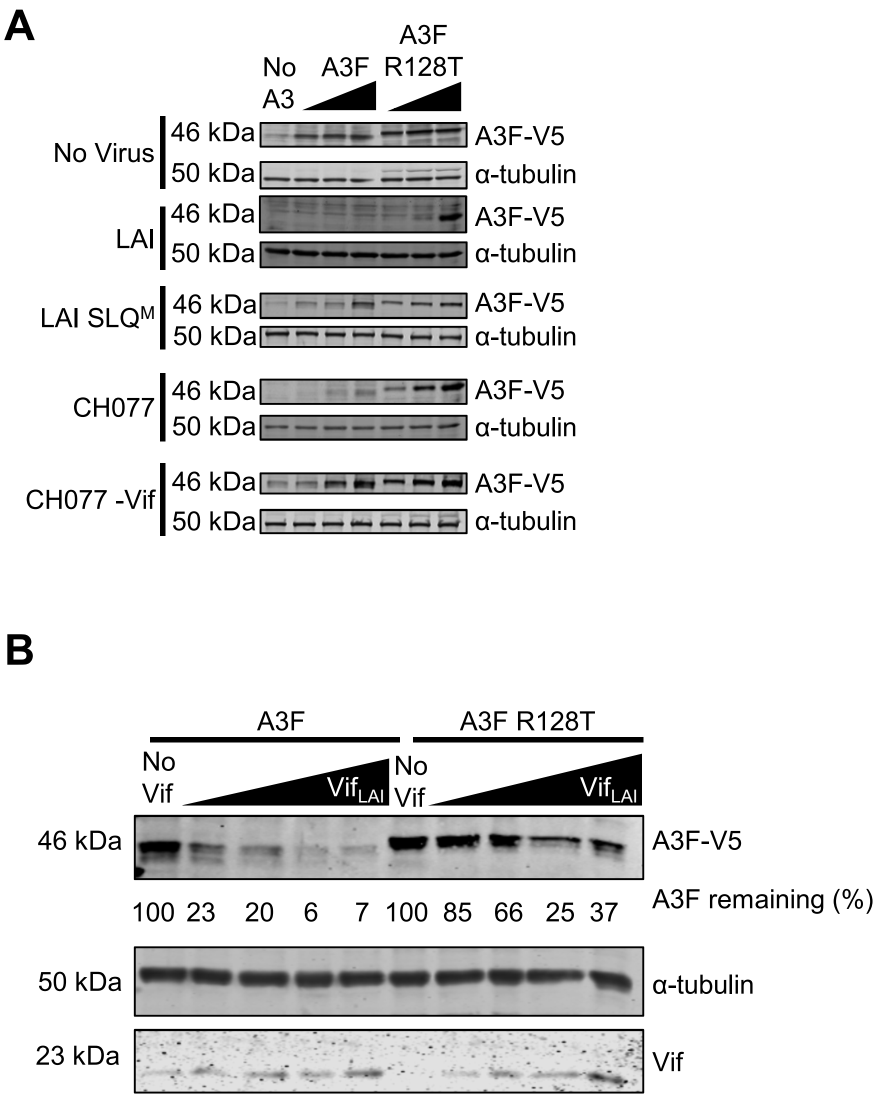

Figure S1. The A3F NTD is a determinant for Vif-mediated degradation. Independent replicate of data shown in Figure 3.

Figure S2

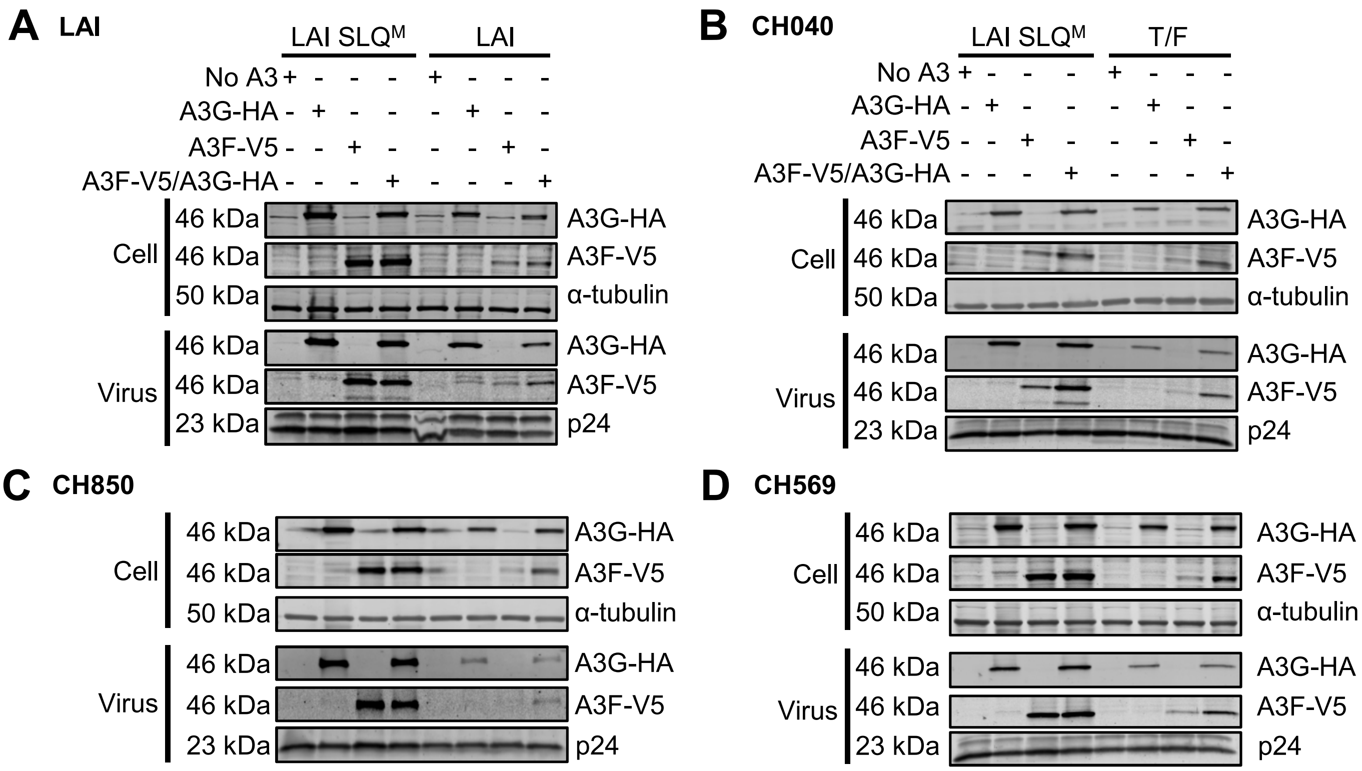

Figure S2. Cell lysates and virus lysates from infectivity experiments showing that HIV-1 T/F viruses have variable infectivity in the presence of A3F, A3G, and A3F/A3G. Independent replicate of data shown in Figure 5.

Figure S3

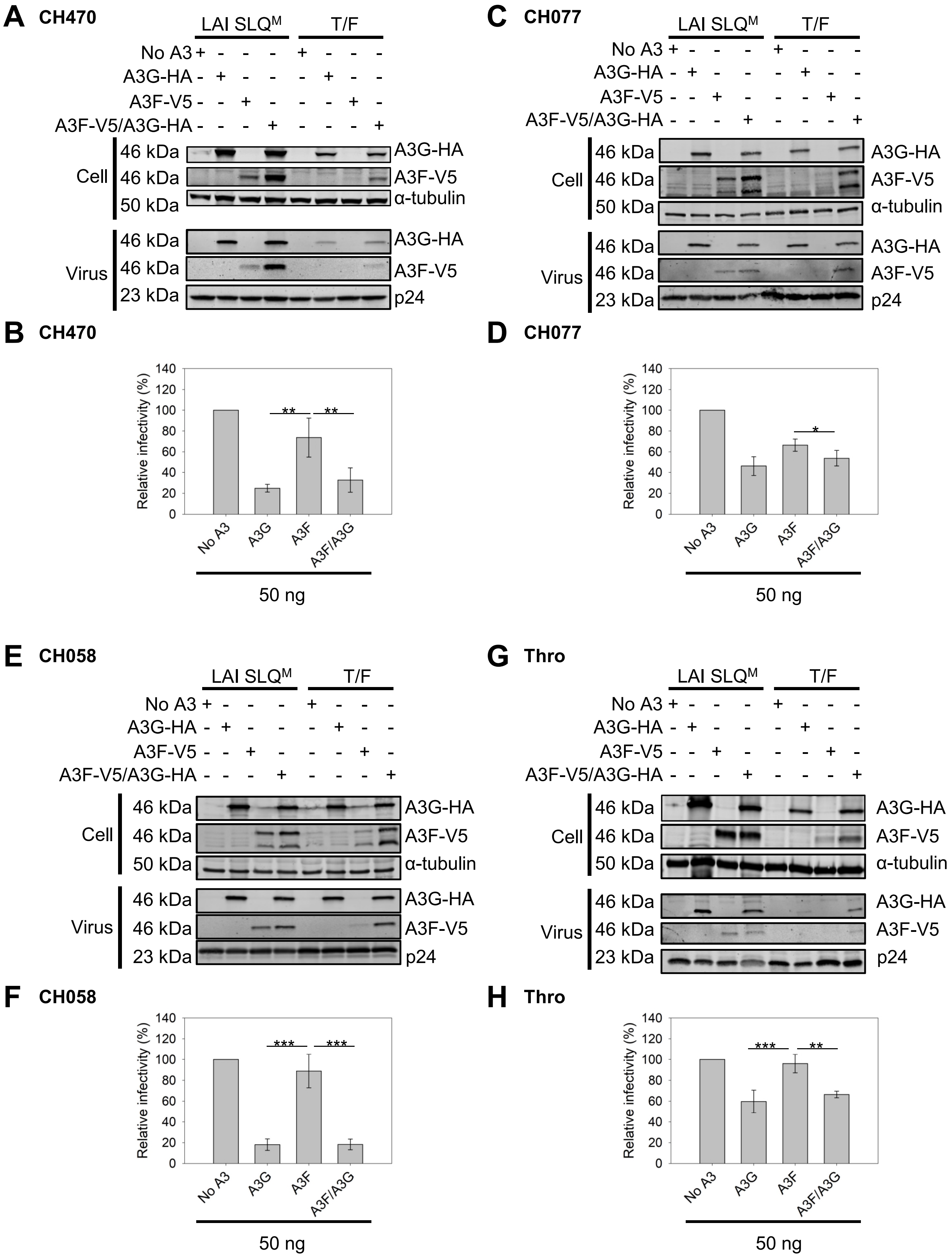

A CH470
LAI SLQ^{M}
T/F
No A3 + - - - + - - -
A3G-HA - + - - - + - -
A3F-V5 - - + - - - + -
A3F-V5/A3G-HA - - - + - - - +
Cell
Virus
46 kDa
50 kDa
23 kDa
A3G-HA
A3F-V5
α-tubulin
p24
B CH470
Relative infectivity (%)
No A3
A3G
A3F
A3F/A3G
50 ng
C CH077
D CH077
E CH058
F CH058
G Thro
H Thro

Figure S3. Subtype B HIV-1 T/F viruses have variable infectivity in the presence of A3F, A3G, and A3F/A3G. 293T cells were co-transfected with No A3 (empty vector), A3G-HA (A3G), A3F-V5 (A3F) or A3F-V5/ A3G-HA (A3F/A3G) expression plasmid and a molecular clone of **(A-B)** HIV-1 T/F CH470 **(C-D)** HIV-1 T/F CH077 **(E-F)** HIV-1 T/F CH058 or **(G-H)** HIV-1 T/F Thro. For immunoblots, cell lysates and virus lysates were collected at 48 h resolved by SDS-PAGE and transferred to nitrocellulose membrane for probing with anti-V5 and anti-HA. The HIV-1_{LAI} SLQ^{M} was used as a no Vif control. The anti-α-tubulin and anti-p24 served as loading control for cell and virus lysate, respectively. For immunoblots, 50 ng of A3 expression plasmid was used. Immunoblots were produced from two independent experiments and showed similar results. The relative infectivity was determined using relative β-galactosidase activity from infected TZM-bl cells normalized to the No A3 condition. Viruses used to infect TZM-bl cells were produced from cells transfected with 50 ng of A3 expression plasmid. Error bars represent the standard deviation from three independent experiments. Designations for significant differences between values were determined using ANOVA and are shown as: $p \leq 0.001$ (***), $p \leq 0.01$(**), or $p \leq 0.05$ (*).

Figure S4

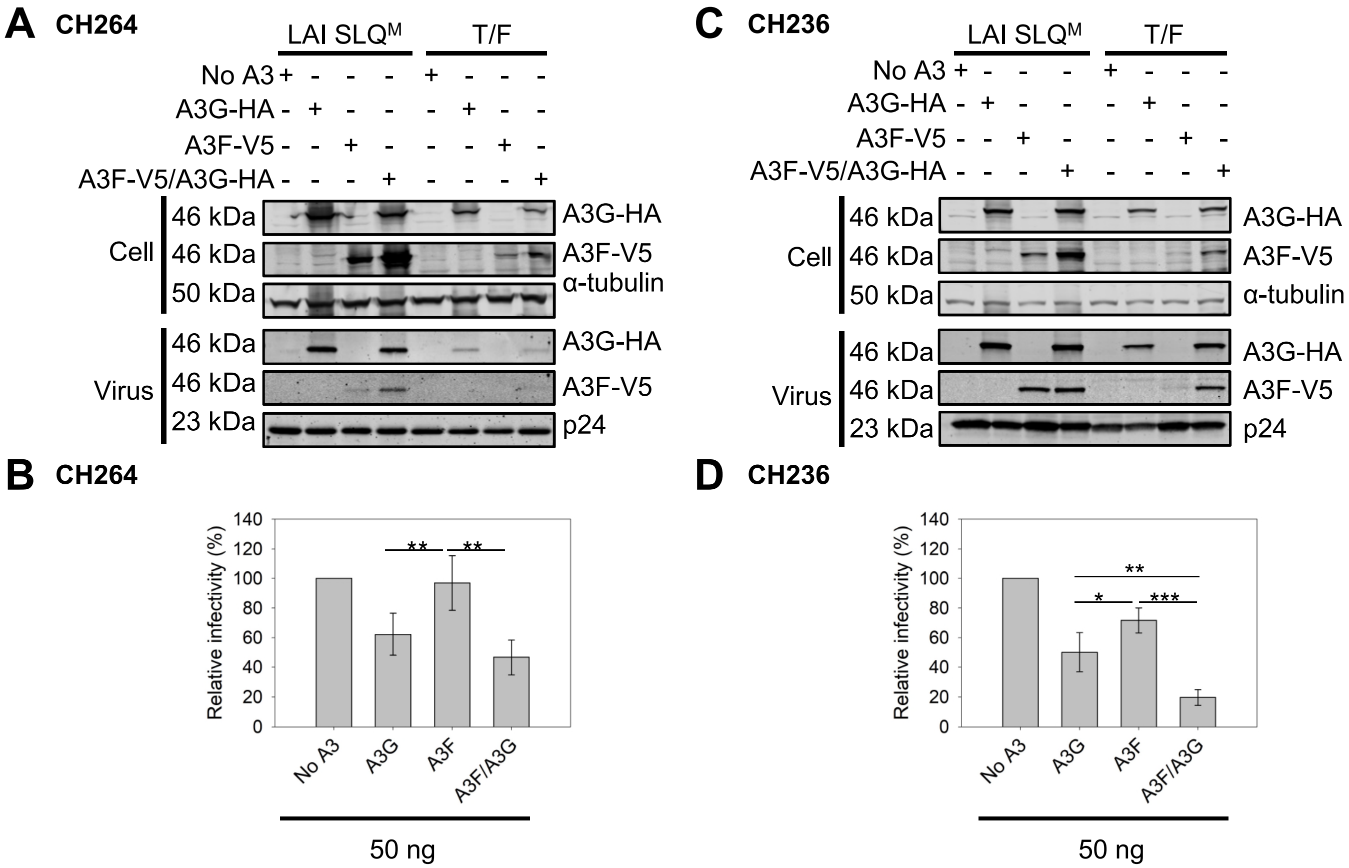

Figure S4. Subtype C HIV-1 T/F viruses have variable infectivity in the presence of A3F, A3G, and A3F/A3G. 293T cells were co-transfected with No A3 (empty vector), A3G-HA (A3G), A3F-V5 (A3F) or A3F-V5/ A3G-HA (A3F/A3G) expression plasmid and a molecular clone of **(A-B)** HIV-1 T/F CH264 **(C-D)** HIV-1 T/F CH236. For immunoblots, cell lysates and virus lysates were collected at 48 h resolved by SDS-PAGE and transferred to nitrocellulose membrane for probing with anti-V5 and anti-HA. The HIV-1_{LAI} SLQ^M was used as a no Vif control. The anti-α-tubulin and anti-p24 served as loading control for cell and virus lysate, respectively. For immunoblots, 50 ng of A3 expression plasmid was used. Immunoblots were produced from two independent experiments and showed similar results. The relative infectivity was determined using relative β-galactosidase activity from infected TZM-bl cells normalized to the No A3 condition. Viruses used to infect TZM-bl cells were produced from cells transfected with 50 ng of A3 expression plasmid. Error bars represent the standard deviation from three independent experiments. Designations for significant differences between values were determined using ANOVA and are shown as: $p \leq 0.001$ (***), $p \leq 0.01$(**), or $p \leq 0.05$ (*).

Figure S5

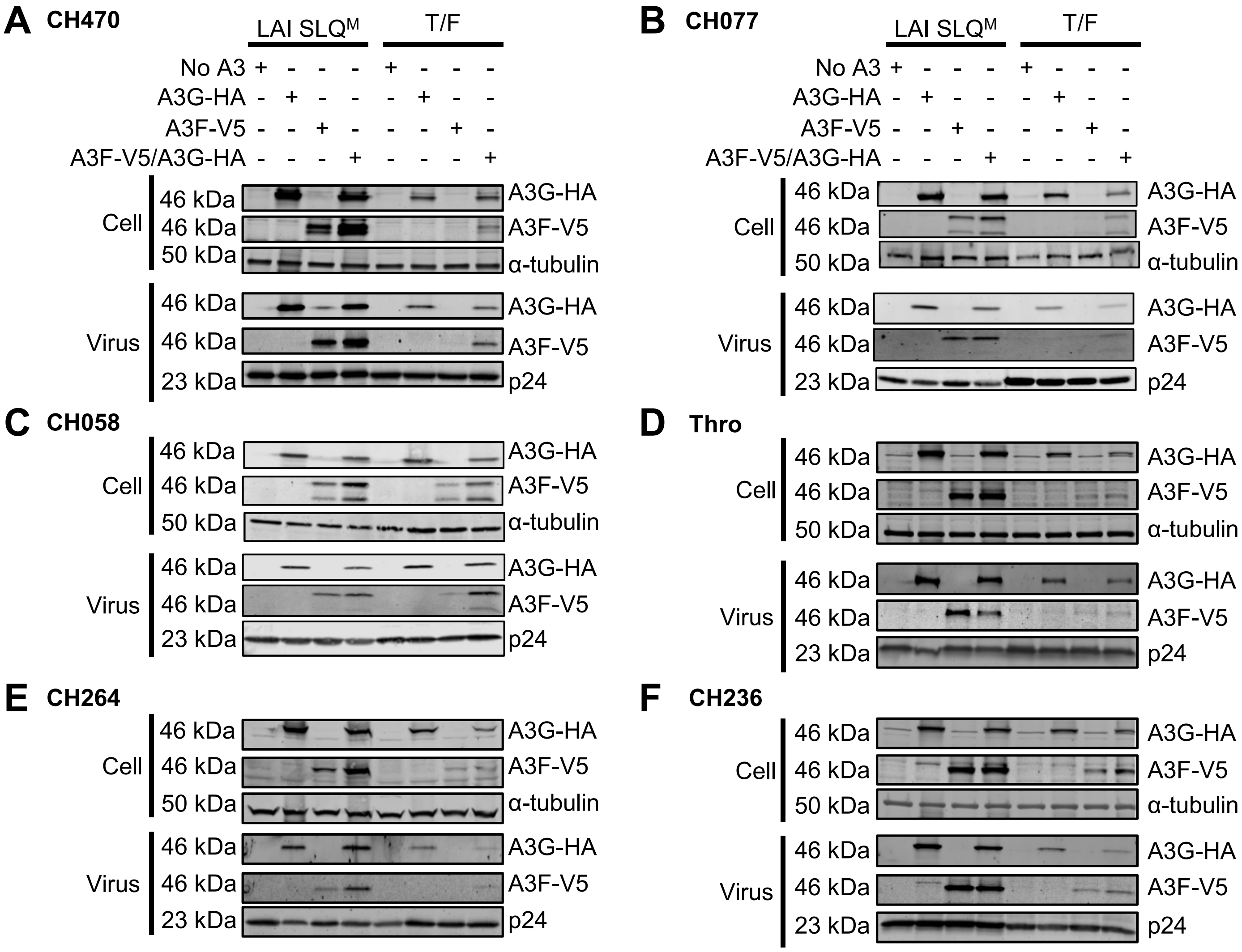

Figure S5. Cell lysates and virus lysates from infectivity experiments showing that HIV-1 T/F viruses have variable infectivity in the presence of A3F, A3G, and A3F/A3G. Independent replicate of data shown in Figure S3 and Figure S4.

Figure S6

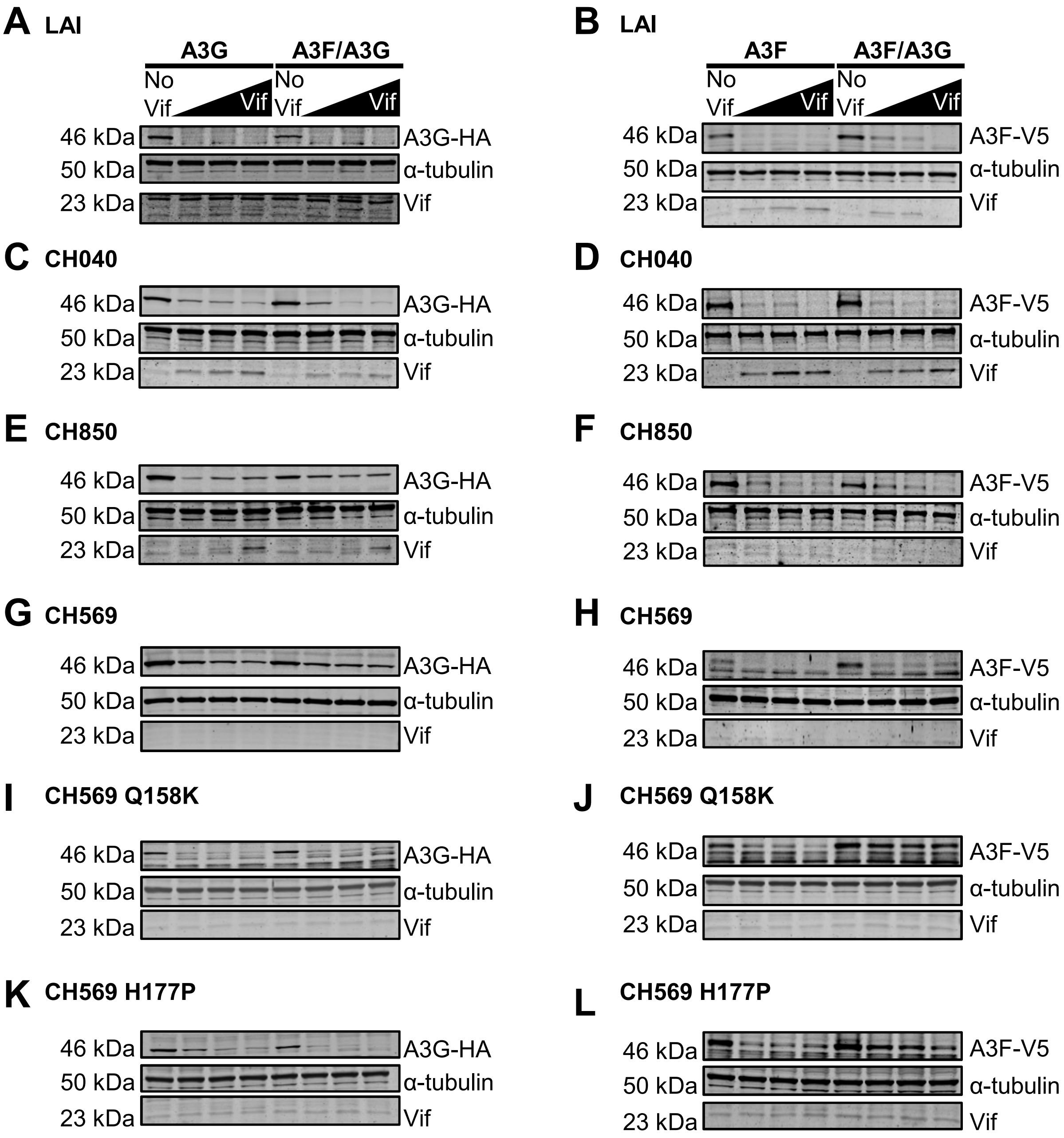

Figure S6. Vif-mediated degradation of A3F, A3G, and A3F/A3G differs between HIV-1 Subtype B and Subtype C viruses. Independent replicate of data shown in Figure 6.
